# The Making of Profit and the British Nurse

**Published:** 1918-07-27

**Authors:** 


### THE MAKING OF PROFIT AND THE BRITISH NURSE.

### The Argument and the Moral.

For the first time on record, so far as I can ascertain, the Economics of Nursing have been discussed by public men (thanks to the *Times*) in the public press. As a free lance correspondent, unfettered by the dogma of any school, may I be permitted to point out that this marks a distinct triumph for The Hospital, inasmuch as the whole subject, including the correspondence, found its origin here? The episode is likewise a triumph for the British Nurse, and marks an epoch in her history. For thirty years, Registration, a subject almost if not altogether academic, has with barren result occupied the attention of the majority. They have been pursuing the shadow, and even the shadow has eluded them. The substance lies hidden in the field of economics, and I hold the belief that in this contention I have the honour of being at one with the Chairman of the "London." Here, however, the road bifurcates. I am not in the unhappy position of being the Chairman of a hospital, and of having to find the money. In the case of a great metropolitan institution the task is a colossal one, and if it devolved on me what Herbert Spencer would have called the bias of responsibility might warp my judgment far more than it has warped the judgment of the Chairman of the "London."

### Is Profit the Object in View?

Lord Knutsford is a dexterous controversialist. He has to be. As he says himself, he has to find the money, and hence his dexterity consists in dodging the issue. But on this occasion I am afraid the Chairman of the "London" has been "winged." It really does not matter whether "Major Dr. Chapple," as Lord Knutsford calls him, followed all the niceties of any particular code of warfare. All that kind of thing belongs to the days of chivalry, and now we fight with gas bombs, under-water bombs and all the rest, so that nobody really cares whether "that honourable and gallant member for Stirlingshire" paid due attention to etiquette, nor indeed is anybody concerned whether Lord Knutsford was irritated or not. These are trifles light as air. The vital issue, and the issue that the Chairman evades, is—Does the London Hospital "farm" out its nurses, or does it not, and if so is the making of profit the object in view?

### Ierne Astonished Beyond Measure.

I must confess that I am astonished beyond measure to see that Lord Knutsford denies the charge. The allegation, he states categorically, is "untrue." When I read this I rubbed my eyes. Am I waking or am I sleeping? I turned up my *Times* (advertisement page) to see if I had been dreaming. I also consulted the Report of the London Hospital, and, finally, I pulled down the dictionary. "To farm" is "to let at a rent": so says the authority on the meaning of words. Major Chapple gives the rent of a "London" nurse as two guineas a week: the Report of the "London" puts it at from two and a half to three and a half. The *Times* by advertisement offers "London" nurses to all who may require their services. And yet Lord Knutsford says the charge is "untrue." As a simple seeker after truth I ask, Where are we? What and whom are we to believe? The conclusion that I have arrived at is that the last word has yet to be uttered, and that it is up to the Chairman of the London Hospital to say that word. Does he employ a staff of nurses to make money for the hospital or not?

### When Ierne Joins the Procession.

I notice that Colonel Maurice suggests that the passing of a Bill to register Certificated nurses will prevent the farming out of the unqualified. With great respect I do not think he is right. No Bill for the Registration of Nurses, except in the case of midwives, has ever proposed to put the untrained woman out of business, and if any Bill such as has been presented to Parliament became law Lord Knutsford could laugh at it. The "London" is still the "London," and with an unregistered staff it could be advertised in the *Times*—"business as usual." There is no use in blinding one's eyes to hard facts. Registration as a protection for the profession is a colossal bogie so long as it fails to prevent women from nursing the sick for gain without the possession of specific qualifications. When it accomplishes this then I join the procession.

### Why Unity has the Maximum of Importance.

I hope I may be permitted to break through the code of newspaper etiquette and point out that in the entire correspondence the most useful note struck was by Sir Henry Burdett, who said "unity has the maximum of importance for British nurses to-day." There is in truth no other sovereign balm for "sweating," or "farming," or "exploiting." And be it remembered that the London Hospital is hardly more a sinner in this respect than any other. They are all guilty of "exploiting" the nurse, whether they rent her out or not, and they do so, not for personal gain, but in order to reduce the cost of running their establishments. All honour to them for high motives. My contention is that the public hospitals of the United Kingdom are for the benefit of the nation, and that the nation ought to pay its workers. I am not out for abusing one man or one set of men. I am out to show that the policy of hospital management is to sacrifice British nurses in the misclaimed interests of philanthropy. It is immoral for the London Hospital to absorb in its revenues the earnings of its nurses, and to regard them as legitimate instruments of profit. I decline to believe that the British people desire to make the nurse the victim of their philanthropy, and hence I come back to the question of union. The College of Nursing stands for union: its Council is an elective Council. Nurses have the power, if they choose to avail themselves of it, of forming and reforming that Council. They can insist on their will and wishes being imposed throughout the realm.

### The Whole Case in a Nutshell.

Let me state a case. Suppose that the College of Nursing could boast of a membership of fifty thousand, which is a not unreasonable approximation of possibility, what is there to prevent the formation by the College of a Private Nurses Co-operation consisting of first-class and well-trained women doing all the business of the Kingdom for their own profit, on the same lines that Lord Knutsford provides such nurses for the benefit of the Whitechapel poor? I have no objection to Lord Knutsford bestowing his own guinea on the poor, but I object to his bestowing mine. Pay me my wage and allow me to dispose of it as I choose. At the same time the London Hospital's method of gathering revenue ought to open the eyes of nurses to the immense possibilities that union and co-operation open up for them. Get together then: let your watchword be union, and let the College of Nursing become in deed and in truth the British Nurses' Parliament.

